# The Measurement of Goal Orientation in Sport: Psychometric Properties of the Polish Version of the Perception of Success Questionnaire (POSQ)

**DOI:** 10.3390/ijerph17186641

**Published:** 2020-09-11

**Authors:** Maciej Tomczak, Małgorzata Walczak, Paweł Kleka, Aleksandra Walczak, Łukasz Bojkowski

**Affiliations:** 1 Department of Psychology, Poznan University of Physical Education, Królowej Jadwigi 27/39, 61-871 Poznań, Poland; walczak@awf.poznan.pl (M.W.); bojkowski@awf.poznan.pl (Ł.B.); 2 Faculty of Psychology and Cognitive Sciences, Adam Mickiewicz University in Poznan, Szamarzewskiego 89, 60-568 Poznań, Poland; pawel.kleka@amu.edu.pl; 3 Faculty of Medicine, Poznan University of Medical Sciences, Fredry 10, 61-701 Poznań, Poland; aleksandra.i.walczak@gmail.com

**Keywords:** goal orientation, psychometric properties, reliability, validity, POSQ

## Abstract

The main aim of the study is a comprehensive assessment of psychometric properties of the Polish version of Perception of Success Questionnaire (POSQ) in sport. Apart from standard psychometric evaluation, the paper presents an analysis of item reliability through the use of Item Response Theory, as well as the analysis of relationships between sport type, level of participation, gender and goal orientation level. The study covered 412 people aged M = 23.46 (SD = 5.40). The Perception of Success Questionnaire (POSQ), the Task and Ego Orientation in Sport Questionnaire (TEOSQ) and the Sport Motivation Scale (SMS-28) were used. High reliability of POSQ ego subscale (α = 0.89, ω = 0.89) and POSQ task subscale (α = 0.90, ω = 0.91) were noted. The test-retest correlations at the two-week interval were ICC = 0.91 for ego subscale, and ICC = 0.71 for task subscale, respectively. Confirmatory factor analysis showed a relatively good fit of the two-factor model to the data (CFI = 0.94). Relationships between the goal orientation measured by the POSQ questionnaire and motivational traits measured by TEOSQ and SMS-28 were obtained. It was also shown that high-performance athletes had higher scores on the ego factor than recreational athletes. Moreover, men had higher scores on the ego factor than women. The Perception of Success Questionnaire (Polish version) is characterized by satisfactory psychometric properties and can be used for scientific research and diagnosis.

## 1. Introduction

Perception of Success Questionnaire (POSQ) was designed to measure goal orientation. It is one of the most frequently cited questionnaires on motivation in sport alongside the Intrinsic Motivation Inventory (IMI), the Sport Motivation Scale (SMS), the Task and Ego Orientation in Sport Questionnaire (TEOSQ), the Situational Motivational Scale (SIMS) and the Behavioral Regulation in Sport Questionnaire (BRSQ) [1]. The POSQ measurement refers to goal orientation which includes both task and ego orientations and is based on Nicholls’ theory. According to this theory, a specific goal orientation is related to one’s unique perception of one’s own abilities and successes, among other things. Task-oriented people perceive success through the possibility of developing their skills. They judge it by the progression of their skills level. They are characterized by perseverance and striving for perfection during performance of tasks. Ego-oriented people, on the other hand, evaluate success through the possibility of being better than someone else. They are oriented to compare their level of performance with other people and are characterized by the will to be the best [2,3,4,5]. In the light of theoretical assumptions, ego and task orientations are supposed to be orthogonal. It is assumed that they result from specific influences on the athlete, where the motivational climate in sports, family or school environment is essential [4].

The development of the Perception of Success Questionnaire began in 1989. First, Roberts and Balague [4] created 48 test items using literature on the perception of success in sport as well as various questionnaires dedicated to measure goal orientation. Expert evaluation of the questions’ adequacy to goal orientation in a sports context was also used. During this process, 29 items were identified, and the obtained version of the questionnaire was tested on students participating in competitive sports. After the analysis of the main components and elimination of some test items, the questionnaire containing 26 questions was obtained. Next, the questionnaire was evaluated in different groups of people participating in sport activities. First, 16 questions with the highest factor loadings were identified, and then, after further research, it was established that six items for each subscale in terms of ego and task orientation would remain. The TEOSQ questionnaire was used to control concurrent validity. High correlations between goal orientation measured both by the POSQ, and the TEOSQ were noted. The Perception of Success Questionnaire was also adapted for children. The two-factor structure obtained initially in exploratory factor analysis was confirmed by using confirmatory factor analysis both for adults and children [4,6,7]. The Perception of Success Questionnaire (POSQ) was validated in other countries, e.g., France, Portugal, Spain, Russia and Turkey [8,9,10,11,12,13].

It has been shown that the goal orientation measured by the Perception of Success Questionnaire (POSQ) was associated with various variables relevant to functioning in the sports area. For example, positive correlations between task orientation and mastery climate, [14] as well as between task orientation and intrinsic motivation [15] were noted. Holgado et al. [16] created predictive models for the task and ego orientations measured by the POSQ questionnaire. The strongest predictors for task orientation were motivational effort, mastery experience and task involvement climate. The strongest predictors for ego orientation were ego normative success, involvement climate, and status/self- achievement. In the review of goal orientation correlations in competitive sport [17], the highest weighted correlation between task subscale measured exclusively with the POSQ questionnaire was observed in case of self-esteem (r = 0.42). On the other hand, the ego subscale obtained the strongest correlation with ego performance/climate (r = 0.35). The authors indicated that correlations might depend on the type of tool (POSQ/TEOSQ) [17]. In turn, a qualitative review of the literature on goal orientation research [18] established that task orientation measured by the POSQ questionnaire is generally higher in individualistic than in collectivistic countries. Besides, high-performance athletes were more goal-oriented than athletes from other groups, although this effect was marginal. Furthermore, men appeared to be more ego-oriented than women [18].

Taking into account the usefulness and popularity of the POSQ questionnaire, the main objective of this paper is to assess comprehensively the psychometric properties of the Polish version of the questionnaire. In addition to the essential psychometric evaluation of reliability and factor structure analysis, the paper presents an analysis of item reliability through the use of Item Response Theory as well as an analysis of the relationship between the type of sport, type of participation, gender and goal orientation.

## 2. Method

### 2.1. Participants

The study covered 412 athletes aged M = 23.46 (SD = 5.40). This group included 208 women and 204 men, 194 professional (high-performance) and 218 recreational athletes, 265 individual and 147 team athletes. The group of women included 92 professional (high-performance) and 116 recreational athletes. The group of men consisted of 102 professional and 102 recreational athletes.

### 2.2. Measurement Tools

In the research, an adapted scale of Perception of Success Questionnaire (POSQ—adult version) by Roberts, Treasure and Balague [4] was used. The scale is designed to measure goal orientation. It contains two subscales, ego and task, and each subscale contains six items. The subject determines on a five-stage scale how much the statement relates to him or her. At first, one of the two linguistic experts translated the questionnaire into Polish. Subsequently the other linguistic expert translated it back into English. The final construct of Polish version of the questionnaire was established by the expert group of three sports psychologists (two of them were also professional trainers) and English translator [19]. Initially, respondents were asked to comment on statements if necessary; however, they had no doubts about understanding the statements. In the opinion of the bioethics—after analyzing the research description—this kind of research does not require the formal consent. (It was not classified as a medical experiment). Each participant was informed in writing that the research is completely anonymous and voluntary and that participation in it constitutes consent.

In order to determine the correlations of both ego and task subscales from the POSQ questionnaire with other motivational components, the TEOSQ questionnaire by Duda [20,21] in the Polish adaptation by Tomczak et al. [5] and Sport Motivation Scale (SMS-28) by Pelletier et al. [22] in the Polish adaptation by Walczak and Tomczak [23] were used. The TEOSQ questionnaire was used to study the goal orientation as it also contains the ego (6 items) and task (7 items) subscales. In both questionnaires, the respondent determines to what extent the statement refers to him or her on a 5-stage scale. Cronbach alpha for the Polish version of TEOSQ was 0.84 for ego subscale and 0.81 for task subscale. Test–retest reliability with two weeks interval was ICC = 0.86 for both subscales [5].

The SMS-28 questionnaire, on the other hand, contains 28 statements that refer to the seven components of motivation according to Self Determination Theory. These include: motivation to know, motivation to accomplish, motivation to experience stimulation, identification, introjection, external regulation and amotivation. The first three components concern intrinsic motivation and the next three extrinsic motivation. The task of the respondent is to determine to what extent a given statement refers to him or her on a 7-stage scale [22]. Reliability of the Polish version was satisfactory, and Cronbach alpha coefficients for each subscale were as follows: motivation to know: 0.81, motivation to accomplish: 0.80, motivation to experience simulation: 0.83, identification: 0.73, introjection: 0.73, external regulation: 0.75, amotivation: 0.77. Test–retest reliability with two weeks interval was between 0.73 for amotivation and 0.83 for experience stimulation [23].

### 2.3. Statistical Analysis

The reliability of the questionnaire was assessed using Cronbach’s alpha (α) and McDonald’s omega (ω) [24]. Moreover, an intraclass correlation [25] was calculated between the results of the same individuals (*n* = 28 athletes, 21 men and 7 women, mean age = 25.14, SD = 9.05) at a two-week interval (test–retest reliability). Additionally, the standard measurement error (SEM), standard measurement error percentage (SEM% = (SEM/mean from means of test and retest) × 100)), smallest real difference (SRD) and smallest real difference percentage (SRD%=(SRD/mean from means of test and retest) × 100) were calculated; see, e.g., [26]. Considering the power of the test 0.8, size of ICC of, e.g., 0.6, 0.7 or 0.8 (as population values), required sample sizes are 15 (for ICC = 0.6), 10 (for ICC = 0.7) and 7 (for ICC = 0.8) [27]. Therefore, a sample size of 28 seems sufficient. The paired t-test was used to compare the mean values in the test and retest.

In order to analyze the reliability of individual test items, the generalized partial credit model (GPCM) scaling from Item Response Theory (IRT) was used. Information function values were given, and information curves were presented. A low information function value for a test item in the context of other values indicates lower reliability of the item due to a higher standard error. The curves show the amount of information provided for a given trait level, which is adequate to the precision of the item in the area of a given trait level. Highly confused and flat information curves show high precision of the position in the whole spectrum of item values. On the other hand, low-placed and flat curves indicate that the position provides little information in the whole spectrum of trait [28,29].

In order to determine the fitting of the theoretical two factorial model assumed by the authors (ego-6-positions/task-6 positions) to empirical data, confirmatory factor analysis was carried out. The model was estimated to comply with the theoretical assumptions without allowing for correlation between the factors. CFI, TLI and NFI values above 0.95 and RMSEA below 0.06 indicate a good fit of the model to the data [30,31]. On the other hand, values above 0.90 often indicate an acceptable fit between model and the data. Values of RMSEA below 0.08 indicate that the model fits the data sufficiently. Moreover, it is sometimes considered that RMSEA values between 0.08–0.10 indicate the mediocre fit, and values above 0.10 indicate the poor fit [32,33]. For power of the test of 0.8 and a close fit test for hypothesis: H_0_: ɛ_0_ = 0.05, H_1_: ɛ_1_ = 0.08 and df = 50, required sample size is 214, while for df = 60, required sample size is 187 [33]. Hence, it can be assumed that the sample size was sufficient.

Additionally, in order to check whether the same construct was measured in women and men, the measurement invariance across gender was carried out. First, it was checked whether the same construct was measured in the groups and the model for configural invariance was tested. Then the differences in the groups of women and men between the factor loadings, intercepts and residuals were tested; models for metric invariance (model with fixed loadings), scalar invariance (model with fixed loadings and intercepts) and strict invariance (model with fixed loadings, intercepts and residuals) were estimated. The fit indexes of each model were compared to the fit indexes of the model estimated before. It was assumed that a change in CFI above 0.01 and a change in RMSEA above 0.015 indicated no measurement invariances in the groups [34].

The r-Pearson correlation was used to assess the relationship between the POSQ goal orientation and other motivational variables (*n* = 331). It was assumed that the correlation 0.1 means a small effect, 0.3 means a medium effect and 0.5 refers to a large effect. For eta square, it was assumed that the value 0.01 is a small effect, 0.06 is a medium effect and 0.14 refers to a large effect [35]. In order to summarize the obtained relations, the canonical analysis was carried out, where ego and task measured by the POSQ were dependent variables, and ego and task measured by TEOSQ questionnaire as well as SMS-28 motivation components were independent variables. During the analysis, so-called canonical variables were created, which were a combination of input dependent and independent variables. Original variables were associated with canonical variables having canonical loadings and weights. Loadings were interpreted similarly to correlations, and weights were interpreted similarly to regression coefficients. The analysis allows for a synthetic approach to the relationship between the two sets of variables [36].

To compare women and men (gender), high-performance and recreational athletes (level of participation) as well as individual and team athletes (sport type) in terms of goal orientation, a three-way Analysis of Variance was applied. Statistical analysis was performed using Statistica Software (13.3 version) and R environment.

## 3. Results

### 3.1. Reliability of the POSQ Questionnaire

Both Cronbach’s alpha and McDonald’s omega for the ego subscale were 0.89, while the item-scale correlations for ego subscale were 1–0.69, 2–0.73, 3–0.75, 6–0.77, 10–0.62, 11–0.70, respectively. For task subscale Cronbach’s alpha was 0.90 and McDonald’s omega was 0.91, while the item-scale correlations for the task subscale were 4–0.60, 5–0.84, 7–0.74, 8–0.82, 9–0.80, 12–0.65, respectively.

### 3.2. Item Response Theory—Reliability of Test Items and POSQ Subscale

**Figure 1:**
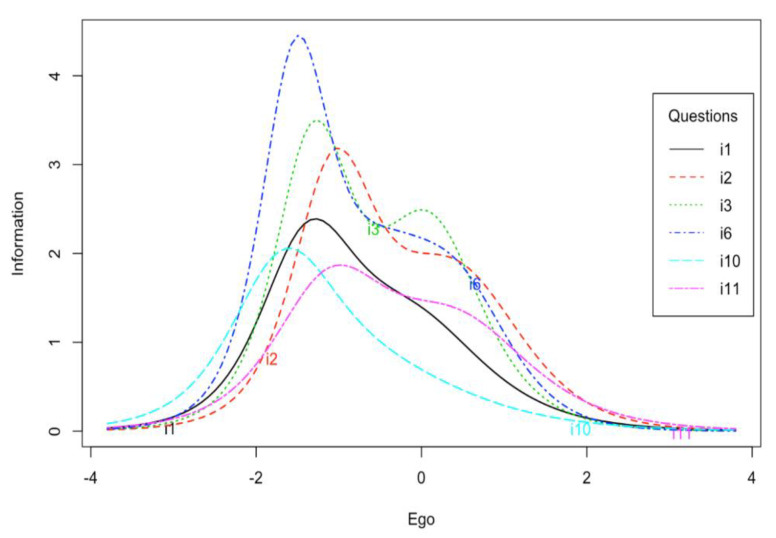
Information curves for the POSQ ego subscale items.

**Figure 2:**
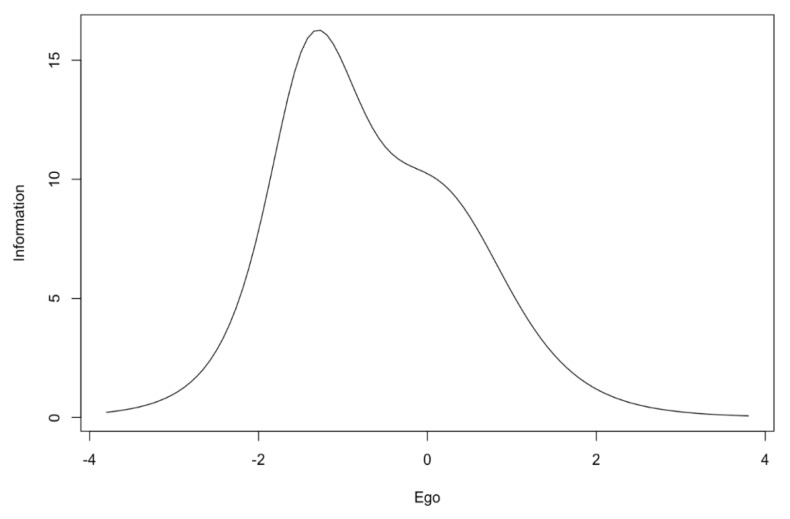
Information curve for the ego subscale.

Very high information function values were obtained for all items (Table 1). Items from the ego subscale provide more information for values that are below the middle of the scale. This statement is especially true for item No. 10, which provides much more information for low level of trait. Other items also provide quite a lot of information for medium level of trait (Figure 1). The subscale generally provides more information for low and medium levels of trait (Figure 2).

**Figure 3:**
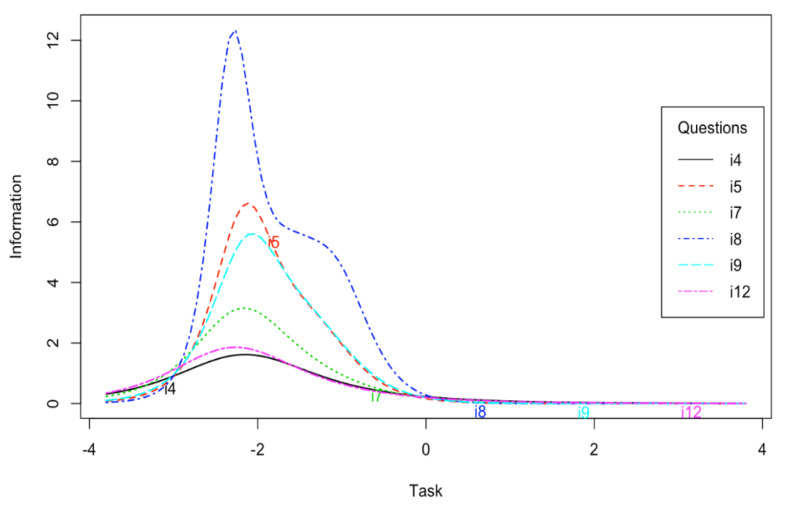
Information curves for the POSQ task subscale items.

**Figure 4:**
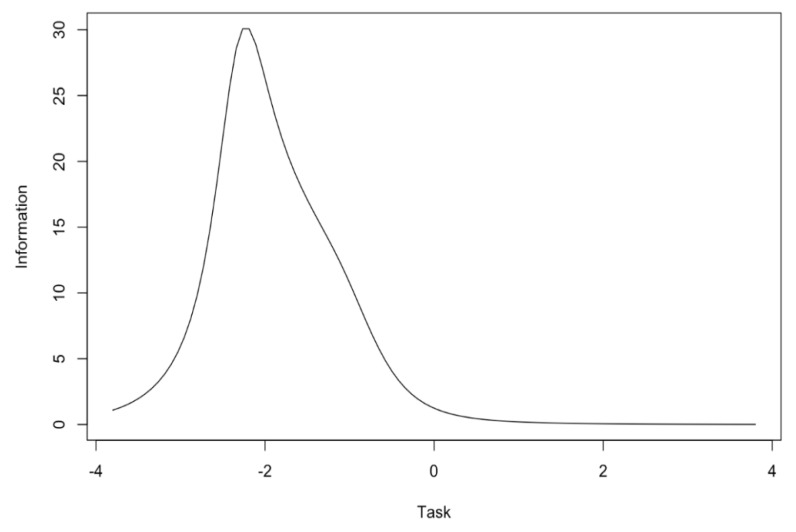
Information curve for the task subscale.

Items for the task subscale provide more information for low level of trait than for medium and high level. Items no 4 and 12 provide less information than other items (Figure 3). The task subscale provides the most information for low level of trait. It discriminates medium and high levels of trait very slightly (Figure 4).

### 3.3. Reliability of the POSQ Assessed by Test-Retest Method

The correlation between the first and second measurements for ego subscale was ICC = 0.91, (95%CI [84, 95], SEM = 0.29, SEM% = 7.60, SRD = 0.80, SRD% = 20.97) and for task subscale ICC = 0.71 respectively (95%CI [51, 84], SEM = 0.20, SEM% = 4.36, SRD = 0.57, SRD% = 12.11). There were no significant differences in the mean scores for the ego subscale in the first and second measurements (Ego1: M = 3.82, SD = 0.94; Ego2: M = 3.81, SD = 1.01; t(27) = 0.08, *p* > 0.05). There were also no significant differences in the mean scores for the task subscale in the first and second measurements (Task1: M = 4.74, SD = 0.34; Task2: M = 4.67, SD = 0.42; t(27) = 1.29, *p* > 0.05).

### 3.4. Confirmatory Factor Analysis of the POSQ Questionnaire

**Figure 5:**
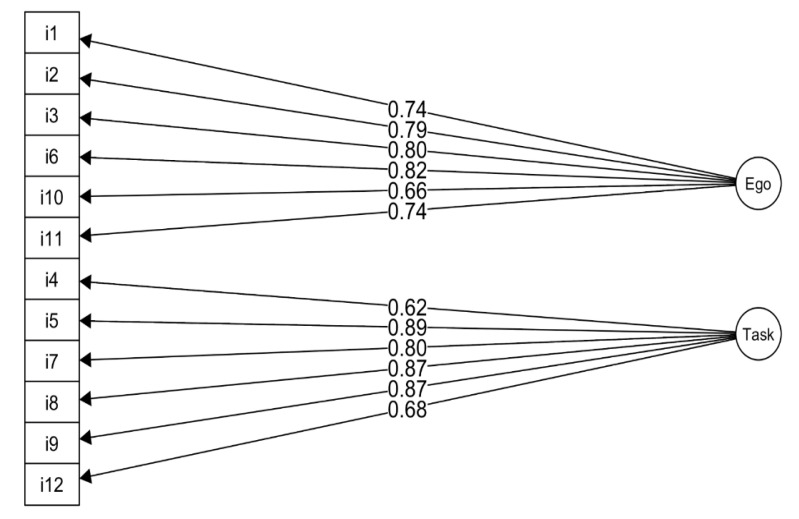
Factors ego and task of the POSQ model: Chi2(54) = 226.86, *p* < 0.001, CFI = 0.942, TLI = 0.929, NFI = 0.926, RMSEA = 0.088 CI.90 [0.076–0.100].

In general, the presented model was relatively well-fitted to the data (Figure 5).

Next, measurement of the invariance across gender for the POSQ questionnaire was conducted (Table 2).

Based on the CFI and TLI indices, the model for the configural invariance obtained an acceptable fit, while the RMSEA value was slightly increased—the value was above 0.08, although it was lower than 0.10. Metric and scalar invariances were confirmed, where ΔCFI, ΔRMSEA were not greater than 0.01. In case of strict invariance, the results of ΔCFI were above the acceptable threshold (0.01), and it indicated lack of invariance across groups, while ΔRMSEA indicated no differences between the models and supported invariance across gender (value below threshold 0.015).

### 3.5. Correlations of the POSQ Goal Orientation with Other Motivational Variables

Relatively strong positive correlation was found between the POSQ ego subscale and the TEOSQ ego subscale. Moreover, positive associations of the POSQ ego subscale with external regulation, amotivation and introjection were noted (Table 3). There was also a significant positive correlation between the POSQ task subscale and the TEOSQ task subscale. Moreover, a positive correlation was noted between the POSQ task subscale and the intrinsic motivation components from the SMS-28 scale (Table 3).

Two significant canonical correlations were obtained. The analysis of factor loadings indicated that the first pair of variables was formed from the ego subscale from the POSQ questionnaire and ego form TEOSQ and external regulation. The second pair of variables was made up of the task subscale from the POSQ questionnaire and the task subscale from the TEOSQ questionnaire along with intrinsic motivation components. The canonical weights were slightly different; however, the biggest difference concerned the second canonical variable. The identification was the most important in the set of independent variables (Table 4).

### 3.6. The Relationship between Sport Type, Level of Participation, Gender and POSQ Subscales

A statistically significant gender effect for ego orientation was observed (Table 5). Men were characterized by higher scores on the ego factor than women (men: M = 3.87, SD = 0.88; women: M = 3.48, SD = 0.97, Table 6). However, this effect was small (η_p_^2^ = 0.03). The main effect of level of participation in sport was also noted (Table 5). High-performance athletes were characterized by higher results on ego subscale than recreational athletes (high-performance athletes: M = 3.97, SD = 0.86; recreational athletes: M = 3.40, SD = 0.94, Table 6). In the remaining cases, the effects were not statistically significant. In the case of task subscale (Table 7 and Table 8), none of the effects were statistically significant.

## 4. Discussion

In the first step, the reliability of the POSQ scale was analyzed. The very high Cronbach’s alpha and McDonald’s omega values were noted. Alpha coefficients were similar or even slightly higher than the values given by authors of the 12-item version of the POSQ as well as by other researchers examining the POSQ questionnaire [4,8,9,11,14]. The reliability determined by the test-retest method was also satisfactory. The correlation for the ego subscale was very high while the correlation for the task subscale was lower but acceptable. The difference between the values of these correlation coefficients might come from differences in the variances of ego and task subscales. The task subscale had a much smaller variance. However, this aspect requires further research. Moreover, the high quality of individual items was indicated by high values of item-rest correlations. The IRT analysis showed that the ego subscale provided more information about low and medium levels of trait. The task subscale, on the other hand, provided more information only for low levels of trait in comparison to medium and high levels of trait. In addition, items 4 and 12 of this subscale provided less information compared to other items. This aspect requires further research to determine to what extent the results obtained are reproducible on other samples.

The analysis of the factor structure showed that the two-factor model adequate for ego and task subscales fits relatively well to empirical data. CFI, TLI, NFI indicators had satisfactory values. The RMSEA index had a slightly increased value. The model presented by authors also obtained not ideal but acceptable fit indices (TLI = 0.90, root mean square = 0.09) [4]. The obtained values indicated that the POSQ questionnaire model was better suited to the data than the model (the first unadjusted model) of the Polish version of the TEOSQ questionnaire presented by the authors in the earlier publication [5]. However, this issue should be treated very carefully because it requires further analysis of the same data samples. Moreover, configural, metric and scalar invariances across gender for the POSQ construct were confirmed. Strict invariance concerning the difference in residuals in the group of men and women requires further research and analysis, as it was not confirmed by change of CFI (however, the RMSEA change was acceptable).

Significant positive relationships were noted between the goal orientations measured by the POSQ and the TEOSQ questionnaires. However, these correlations were weaker than reported by the authors of the original questionnaire [4]. In particular, there was a weaker correlation between the task subscales from both tools. On the other hand, the obtained correlation coefficient for task subscales was similar to the value obtained by Fogarty et al. [37], which was only slightly higher. The correlation between the POSQ and TEOSQ ego subscales was relatively strong. Moreover, significant positive correlations were obtained between the task orientation and the components of intrinsic motivation determined by the SMS-28. The directions of these relations were consistent with the predictions, although they were not very strong. These aspects may indicate a moderating role of both tools (POSQ, TEOSQ) [17]. The relationships between the task orientation measured by the TEOSQ questionnaire and the intrinsic motivation components (SMS-28) presented in the publication which concerned the Polish version of the TEOSQ questionnaire were more than twice as high [5]. On the other hand, the relationship between the POSQ task orientation and intrinsic motivation was not always clear. For example, Gomez-Lopez et al. [38] did not demonstrate a significant relationship between these variables when studying basketball players; however, positive relationships between ego orientation and external regulation were noted, which is consistent with the previous studies [17]. Those associations were also confirmed in a comprehensive canonical analysis of the Polish version of the POSQ questionnaire. This analysis showed that for ego orientation assessed by the POSQ, the most important predictors was ego orientation measured by the TEOSQ questionnaire and external regulation (SMS-28). In turn, for the task orientation measured by the POSQ questionnaire, the most important predictors were the task orientation assessed by the TEOSQ questionnaire and intrinsic motivation components (SMS-28). The picture is slightly different in the analysis of canonical weights, where for the second canonical variable, the identification was the most important. However, this effect could result from, e.g., high collinearity of independent variables.

The analysis of intergroup comparisons showed that men had higher scores on the ego factor than women. However, this effect despite its statistical significance was not too high. This effect was consistent with the previously presented study results and usually occurs while using the POSQ questionnaire but does not apply to the TEOSQ questionnaire [18]. In the study on the validation of the Polish version of the TEOSQ questionnaire, no difference between men and women was noted [5]. The validation of the Polish version of the POSQ questionnaire showed that recreational athletes had lower scores on ego scale than professional athletes. Therefore, a higher self-focus may be propitious for adapting to a sports situation in which the result obtained is usually essential. However, this issue is not clear, e.g., this effect did not occur in the case of TEOSQ goal orientation. The analysis of the Polish version of the POSQ questionnaire did not indicate significant effects for the task subscale.

Study and analysis had some limitations. Despite the acceptable ICC values for reliability tested by the test–retest method, in this case, an interesting discrepancy between the correlation value for the ego and task subscales was observed. The value for the task subscale was much lower. It is difficult to clearly establish where it came from. Hence, it is worth to control the test–retest reliability, e.g., on a larger and more homogeneous group. The IRT analysis showed that items 4 and 12 provided less information than the other items. In addition, the task subscale provided more information on the low trait levels. It is difficult to clearly explain the reason for it in this case. On the other hand, although the fit of the CFA model was acceptable based on CFI, TLI, NFI, the RMSEA values were a bit too high. Testing the model on other samples is worth considering. Additionally, it has been established that there is a configural, metric and scalar invariances across gender. However, neither confirmation nor consistent result was obtained for strict invariance, and it also requires further research and analysis.

The paper presents and discusses the psychometric properties of the Polish version of the POSQ questionnaire. Subsequent analyses may concern the comparison of psychometric properties and relations between different variables measured by the POSQ and the TEOSQ questionnaires on the same samples. In line with previous analyses [17], it seems that despite significant conceptual and empirical similarities, there are also important differences resulting from the measurement of the POSQ and the TEOSQ questionnaires.

## 5. Conclusions

The Polish version of the Perception of Success Questionnaire for adults is characterized by satisfactory psychometric properties and can be used for scientific and diagnostic purposes. Additionally relations between level of participation, gender and goal orientation were observed, i.e., high-performance athletes were characterized by higher results on ego subscale than recreational athletes. Men were characterized by higher scores on the ego factor than women; however, this effect was small.

## Figures and Tables

**Table 1:** The values of information function for Perception of Success Questionnaire (POSQ) test items.

Items	InfoRange	PropRange
i1	5.8085	0.9963
i2	7.5524	0.9987
i3	8.0636	0.9991
i6	9.3164	0.9993
i10	4.7463	0.9895
i11	5.7651	0.9944
i4	3.5655	0.9464
i5	8.8501	0.9977
i7	5.3797	0.9802
i8	15.7083	0.9995
i9	9.5439	0.9987
i12	4.1275	0.9575

**Table 2:** Invariance measurement across gender for the POSQ questionnaire.

Invariance	CFI	TLI	RMSEA	ΔCFI	ΔRMSEA
Configural (1)	0.939	0.926	0.091	-	-
Metric (2)	0.935	0.927	0.090	0.004	0.001
Scalar (3)	0.931	0.929	0.089	0.004	0.001
Strict (4)	0.912	0.917	0.096	0.019	0.007

ΔCFI, ΔRMSEA—differences between nested models: 1–2, 2–3, 3–4.

**Table 3:** Correlations of POSQ task subscale and ego subscale with the TEOSQ goal orientation and the SMS-28 motivation components.

Orientation POSQ	*n* = 331							
	E/T	KN	AC	ES	IDF	INT	ER	AMT
EGO	0.59 *p* < 0.001	−0.09 *p* = 0.110	−0.05 *p* = 0.393	0.07 *p* = 0.193	0.06 *p* = 0.257	0.12 *p* = 0.035	0.40 *p* < 0.001	0.17 *p* = 0.002
TASK	0.25 *p* < 0.001	0.22 *p* < 0.001	0.21 *p* < 0.001	0.15 *p* = 0.005	0.01 *p* = 0.919	0.05 *p* = 0.412	−0.10 *p* = 0.073	−0.09 *p* = 0.107

KN—intrinsic motivation to know; AC—intrinsic motivation to accomplish; ES—intrinsic motivation to experience stimulation; IDF—identification; INT—introjection; ER—external regulation; AMT—amotivation; E/T—upper field: correlation between the POSQ ego subscale and the TEOSQ ego subscale; lower field: correlation between the POSQ task subscale and the TEOSQ task subscale.

**Table 4:** Characteristics of canonical variables.

Variables	Canonical Variables			
	1		2	
	Loadings	Weighs	Loadings	Weighs
**EGO**	−0.89	−0.96	−0.46	−0.32
**TASK**	0.32	0.46	−0.95	−0.90
**Canonical correlation**	0.70 (*p* < 0.001)		0.25 (*p* < 0.01)	
**Variables**	**1**		**2**	
**E**	−0.89	−0.77	−0.30	−0.29
**T**	0.37	0.34	−0.71	−0.43
**KN**	0.27	0.13	−0.69	−0.43
**AC**	0.20	0.08	−0.70	−0.28
**ES**	0.00	−0.19	−0.64	−0.24
**IDF**	−0.08	0.00	−0.10	0.53
**INT**	−0.13	0.00	−0.31	−0.05
**ER**	−0.61	−0.23	−0.16	−0.10
**AMT**	−0.29	0.01	0.10	−0.18

**Table 5:** Three-way ANOVA summary for the POSQ ego subscale.

Effect	Df	F	*p*	Partial Eta Square
Intercept	1	6062.15	0.0000	0.94
gender	1	13.59	0.0003	0.03
participation	1	32.04	0.0000	0.07
sport type	1	0.97	0.3259	0.00
gender * participation	1	0.87	0.3513	0.00
gender * sport type	1	0.22	0.6409	0.00
participation * sport type	1	0.28	0.5943	0.00
gender * participation * sport type	1	0.01	0.9223	0.00
Error	404			

Gender—men, women; participation—high-performance, recreational; sport type—individual, team.

**Table 6:** Descriptive statistics for ego subscale.

Factor Level	Factor Level	Factor Level	*n*	EGO Mean	EGO S.D.	EGO S.E.
			412	3.67	0.94	0.05
Male			204	3.87	0.88	0.06
Female			208	3.48	0.97	0.07
High-performance			194	3.97	0.86	0.06
Recreational			218	3.40	0.94	0.06
Individual			265	3.58	0.98	0.06
Team			147	3.84	0.86	0.07
Male	High-performance		102	4.18	0.71	0.07
Male	Recreational		102	3.55	0.91	0.09
Female	High-performance		92	3.73	0.95	0.10
Female	Recreational		116	3.27	0.94	0.09
Male	Individual		123	3.78	0.94	0.08
Male	Team		81	4.00	0.76	0.08
Female	Individual		142	3.40	0.98	0.08
Female	Team		66	3.64	0.95	0.12
High-performance	Individual		101	3.88	0.91	0.09
High-performance	Team		93	4.06	0.81	0.08
Recreational	Individual		164	3.39	0.97	0.08
Recreational	Team		54	3.45	0.82	0.11
Male	High-performance	Individual	49	4.14	0.79	0.11
Male	High-performance	Team	53	4.23	0.64	0.09
Male	Recreational	Individual	74	3.55	0.96	0.11
Male	Recreational	Team	28	3.56	0.78	0.15
Female	High-performance	Individual	52	3.64	0.95	0.13
Female	High-performance	Team	40	3.84	0.96	0.15
Female	Recreational	Individual	90	3.26	0.97	0.10
Female	Recreational	Team	26	3.33	0.86	0.17

S.D.—Standard deviation, S.E.—Standard error.

**Table 7:** Three-way ANOVA summary for the POSQ task subscale.

Effect	Df	F	*p*	Partial Eta Square
Intercept	1	24,137.00	0.0000	0.98
gender	1	2.22	0.1372	0.00
participation	1	0.13	0.7189	0.00
sport type	1	1.17	0.2806	0.00
gender * participation	1	2.37	0.1242	0.01
gender * sport type	1	3.51	0.0616	0.01
participation * sport type	1	0.76	0.3834	0.00
gender * participation * sport	1	0.47	0.4912	0.00
Error	404			

Gender—men, women; participation—high-performance, recreational; sport type—individual, team.

**Table 8:** Descriptive statistics for task subscale.

Factor Level	Factor Level	Factor Level	*n*	TAS Mean	TASK S.D.	TASK S.E.
			412	4.73	0.57	0.03
Male			204	4.70	0.51	0.03
Female			208	4.76	0.62	0.04
High-performance			194	4.70	0.63	0.04
Recreational			218	4.75	0.51	0.03
Individual			265	4.75	0.58	0.04
Team			147	4.68	0.55	0.04
Male	High-performance		102	4.70	0.51	0.05
Male	Recreational		102	4.69	0.51	0.05
Female	High-performance		92	4.70	0.74	0.08
Female	Recreational		116	4.80	0.52	0.05
Male	Individual		123	4.76	0.47	0.04
Male	Team		81	4.60	0.54	0.06
Female	Individual		142	4.75	0.66	0.05
Female	Team		66	4.77	0.54	0.07
High-performance	Individual		101	4.71	0.72	0.07
High-performance	Team		93	4.69	0.52	0.05
Recreational	Individual		164	4.78	0.48	0.04
Recreational	Team		54	4.66	0.60	0.08
Male	High-performance	Individual	49	4.74	0.63	0.09
Male	High-Performance	Team	53	4.66	0.37	0.05
Male	Recreational	Individual	74	4.77	0.34	0.04
Male	Recreational	Team	28	4.49	0.77	0.14
Female	High-performance	Individual	52	4.67	0.79	0.11
Female	High-performance	Team	40	4.73	0.66	0.10
Female	Recreational	Individual	90	4.80	0.57	0.06
Female	Recreational	Team	26	4.83	0.25	0.05

S.D.—Standard deviation, S.E.—Standard error.
